# Cross-Neutralizing Anti-Chikungunya and Anti-Dengue 2 IgG Antibodies from Patients and BALB/c Mice against Dengue and Chikungunya Viruses

**DOI:** 10.3390/v16071098

**Published:** 2024-07-08

**Authors:** Araceli Posadas-Mondragón, José Angel Santiago-Cruz, Angélica Pérez-Juárez, Norma Estela Herrera-González, Sara M. Sosa-Delgado, Claudia Elena Wong-Arámbula, Abril Paulina Rodríguez-Maldonado, Mauricio Vázquez-Pichardo, Daniel Duran-Ayala, José Leopoldo Aguilar-Faisal

**Affiliations:** 1 Laboratorio de Medicina de Conservación de la Sección de Estudios de Posgrado e Investigación, Escuela Superior de Medicina, Instituto Politécnico Nacional, Mexico City 11340, Mexico; aposadasm@ipn.mx (A.P.-M.); jsantiagoc@ipn.mx (J.A.S.-C.); aperezj@ipn.mx (A.P.-J.); nherrerag@ipn.mx (N.E.H.-G.); ssosad@ipn.mx (S.M.S.-D.); 2 Laboratorio de Ecología Microbiana, Escuela Nacional de Ciencias Biológicas, Instituto Politécnico Nacional, Mexico City 11340, Mexico; 3 Laboratorio de Genoma de Patógenos, Instituto de Diagnóstico y Referencia Epidemiológicos (InDRE) “Dr. Manuel Martínez Báez”, Secretaría de Salud, Mexico City 01480, Mexico; claudia.wong@salud.gob.mx (C.E.W.-A.); abril.rodriguez@salud.gob.mx (A.P.R.-M.); 4 Laboratorio de Arbovirus, Instituto de Diagnóstico y Referencia Epidemiológicos (InDRE) “Dr. Manuel Martínez Báez”, Secretaría de Salud, Mexico City 01480, Mexico; mauricio.vazquez@salud.gob.mx (M.V.-P.); daniel.duran@salud.gob.mx (D.D.-A.)

**Keywords:** dengue virus, Chikungunya virus, cross-reactivity, neutralizing capacity, co-infection, arbovirus, *Flavivirus*, *Alphavirus*

## Abstract

Dengue (DENV) and Chikungunya (CHIKV) viruses can be transmitted simultaneously by *Aedes* mosquitoes, and there may be co-infections in humans. However, how the adaptive immune response is modified in the host has yet to be known entirely. In this study, we analyzed the cross-reactivity and neutralizing activity of IgG antibodies against DENV and CHIKV in sera of patients from the Mexican Institute of Social Security in Veracruz, Mexico, collected in 2013 and 2015 and using IgG antibodies of BALB/c mice inoculated with DENV and/or CHIKV. Mice first inoculated with DENV and then with CHIKV produced IgG antibodies that neutralized both viruses. Mice were inoculated with CHIKV, and then with DENV; they had IgG antibodies with more significant anti-CHIKV IgG antibody neutralizing activity. However, the inoculation only with CHIKV resulted in better neutralization of DENV2. In sera obtained from patients in 2013, significant cross-reactivity and low anti-CHIKV IgG antibody neutralizing activity were observed. In CHIKV-positive 2015 sera, the anti-DENV IgG antibody neutralizing activity was high. These results suggest that CHIKV stimulates DENV2-induced memory responses and vice versa. Furthermore, cross-reactivity between the two viruses generated neutralizing antibodies, but exchanging CHIKV for DENV2 generated a better anti-CHIKV neutralizing response.

## 1. Introduction

Dengue (DENV) and Chikungunya (CHIKV) viruses can cause periodic epidemic outbreaks, particularly in densely populated tropical urban areas. DENV and CHIKV are arboviruses whose genomes are single-stranded RNA of positive polarity [1,2]. One of the most pathogenic *Flavivirus* in humans is dengue virus (DENV), which exists in four serotypes [3]. Chikungunya virus (CHIKV) belongs to the genus *Alphavirus*, family *Togaviridae* [2]. In 2013, the first cases of Chikungunya were reported in the Americas, and the first autochthonous case in Mexico was reported in the State of Chiapas in October 2014 [4,5]. *Aedes aegypti* and *Aedes albopictus* mosquitoes transmit DENV and CHIKV; both vectors are widely distributed in tropical and subtropical regions [6]. The DENV E consists of three domains: a central β-barrel (domain I), an elongated dimerization region (domain II), and a C-terminal immunoglobulin-like region (domain III). ED3 contains the critical epitopes recognized by neutralizing antibodies [7]. Following primary DENV infection, patients generate antibodies that bind to specific EDIII serotype epitopes, and in secondary infection, they produce antibodies against cross-reactive epitopes in EDIII [8]. However, antibodies against EDIII epitopes represent a minor fraction of all anti-DENV serum antibodies [8]. In addition, secondary infections with different serotypes due to the simultaneous circulation of four dengue serotypes (hyperendemicity) result in antibody-dependent enhancements due to the presence complexes of virions with the DENV-specific antibodies via Fc-fragment of IgG in cells with Fcγ receptors (FcγRs) as monocytes, macrophages, dendritic cells, B lymphocytes, natural killer cells, neutrophils, eosinophils, basophils, and platelets, which enhance the internalization of the virus but do not neutralize it [9,10,11]. *Flavivirus* infections generally present similar clinical characteristics, such as fever, rash, conjunctivitis, headache, fatigue, hemorrhagic fever, vascular permeability, encephalitis, biphasic fever, flaccid paralysis, and jaundice [12]; the co-occurrence of different *Flaviviruses* in different geographical areas is common [12]. Infections with *Flavivirus* induce cross-reactive antibodies [7]. Between 15.4% and 84% of the antibodies produced against non-DENV *Flaviviruses* were reported cross-reactivity with dengue virus [13]. It was reported that 6% of the antibodies produced against DENV and 7% produced against non-DENV *Flavivirus* have cross-reactivity with CHIKV (*Alphavirus*) [14].

CHIKV–DENV co-infections have been widely described [15,16]. However, it is not entirely known how the host’s adaptive immune response is modified when these co-infections or primary DENV infections and subsequent CHIKV infections occur, and vice versa.

Therefore, the objective of this study was to analyze the neutralizing capacity of anti-DENV2 and anti-CHIKV IgG antibodies against CHIKV and DENV, respectively, in sera obtained from patients from Veracruz, Mexico, during the epidemiological outbreaks of 2013 (no human cases of CHIKV were reported, only DENV) and 2015 (CHIKV and DENV cases reported in humans), as well as in sera from BALB/c mice inoculated subcutaneously with DENV2 and/or CHIKV.

In serum samples from 2013 patients, when there were no reported chikungunya cases in Mexico, we found that anti-DENV IgG antibodies cross-reacted with CHIKV and had neutralizing capacity. In the same way, in serum samples from patients from 2015, when there were reported cases of CHIKV in Mexico, we found that serums that were positive for anti-CHIKV IgG antibodies cross-reacted with DENV and had neutralizing capacity. In a BALB/c murine model, we found that IgG antibodies produced by mice inoculated with DENV2 reacted with CHIKV (cross-reactivity). Similarly, IgG antibodies produced by mice inoculated with CHIKV reacted with DENV2. This cross-reaction was more evident when the mice were first inoculated with CHIKV and afterward with DENV2. This last group of mice produced a higher titer of anti-CHIKV IgG antibodies with neutralizing capacity; this was even observed in the group of mice that were only inoculated with DENV2, which presented a stronger IgG cross-reactive and neutralizing capacity with CHIKV. These results showed that cross-reactivity between both viruses generated neutralizing antibodies, but the exchange of CHIKV for DENV2 yielded a better neutralizing anti-CHIKV antibody response.

## 2. Materials and Methods

### 2.1. Human Blood Samples

Sera from patients over 18 years old were collected during the 2013 and 2015 epidemic outbreaks in the Family Medicine Unit No. 61 and General Hospital No. 71 of the Mexican Social Security Institute in Veracruz, Mexico. Samples were obtained during the acute phase of the disease (1–8 days after symptom onset). The dengue classification was made considering the WHO criteria in 1997 [17]: for dengue fever (DF), platelet count > 100 × 10^9^/L, and for dengue hemorrhagic fever, platelet count < 100 × 10^9^/L in addition to any hemorrhagic manifestation. The diagnosis of dengue and Chikungunya was confirmed by ELISA and RT-PCR, as described below. The patient provided informed consent. This study was approved by the National Commission of Scientific Research of the Mexican Social Security Institute (Registration No. 2010-785-041) and by the Local Committee for Research and Ethics in Health Research Unit No. 3003 of Family Medicine Unit No. 60 of Veracruz (Registration No. 2810-009-018).

### 2.2. Cells

*Aedes albopictus* C6/36-HT cells were cultured in Minimum Essential Media (MEM; Sigma, Marlborough, MA, USA) supplemented with 5% fetal bovine serum and 100 units/mL of penicillin/streptomycin (L0022, Biowest, Nuaillé, France); cells were grown at 35 °C. Vero E6 and Vero cells were cultured using RPMI-1640 (P0870, Biowest) and DMEM-F12 (12500062, Gibco, Grand Island, NY, USA) respectively, supplemented with 5% fetal bovine serum and 100 units/mL of penicillin/streptomycin (L0022, Biowest); both cell lines were cultured at 37 °C with 5% CO_2_.

### 2.3. Viruses

Reference strains DENV-1 Hawaii, DENV-2 New Guinea C, DENV-3 H-87, and DENV-4 H-241 were donated by Dr. Juan Santiago Salas Benito from the National School of Medicine and Homeopathy of the National Polytechnic Institute [18].

The chikungunya virus used in this study was hChik/Mexico/YUC_InDRE_10815/2015, III-Asian genotype (EPI_ISL_19196361, GISAID). It was isolated in Vero cells from the serum of a patient from Yucatán, Mexico in 2015. CHIKV detection was performed in the Virological Diagnostic Laboratory of the National Institute of Respiratory Diseases and for our laboratory by RT-PCR, as described below. The virus sequencing was developed in the Arbovirus Laboratory and Pathogen Genome Laboratory of the Institute for Epidemiologic Diagnosis and Reference (InDRE). In Mexico, only the Asian genotype circulated during 2014 and 2015 [19,20].

C6/36-HT cells were infected with DENV-2 New Guinea C and Chikungunya virus. The supernatant was harvested until a cytopathic effect was observed. Both viruses were purified with 40% polyethylene glycol (PEG) in sodium chloride at a ratio of 10 mL of PEG for each 40 mL of supernatant; the mixture was left at 4 °C overnight. It was centrifuged at 10,000 rpm for 60 min at 4 °C; the obtained pellet was dissolved in a proportion 1/15 of the original volume with TGNE buffer (Tris 50 mM, glycine 200 mM, NaCl 100 mM, EDTA 1 mM). Aliquots of purified virus were stored at −70 °C. Viral titer was quantified in Vero cells using the plate-limiting dilution technique and in protein concentration using the Bradford method.

### 2.4. Detection of Dengue and Chikungunya Viruses in Sera Patients

RNA was isolated from human sera with the QIAamp Viral RNA mini kit (52906, QIAGEN, Germantown, MD, USA). Subsequently, DENV was identified via multiplex quantitative RT-qPCR. Primers comprising Flavivirus and four fluorogenic TaqMan probes specific for each serotype of DENV in the NS5 region were used [21]. Chikungunya virus was identified via RT-qPCR with the primers CHIKV 6856: 5′ TCACTCCCTGTTGGACTTGATAGA 3′ and CHIKV 6981: 5′ TTGACGAACAGAGTTAGGAACATACC 3, fluorogenic TaqMan probe CHIKV 6919-FAM: 5′ AGGTACGCGCTTCAAGTTCGGCG 3′ [22]. A Superscript III Platinum enzyme One-Step RT-qPCR Kit (1732088; Invitrogen, Thermofisher, Austin, TX, USA) was used for this process. The analysis was performed in a LightCycler 480 instrument (Roche, Indianapolis, IN, USA). DENV-1 Hawaii, DENV-2 New Guinea C, DENV-3 H-87, and DENV-4 H-241 strains were used as positive controls.

### 2.5. Detection of Anti-Dengue and Anti-Chikungunya IgM and IgG Antibodies by Capture ELISA in Sera Patients

IgM and IgG antibodies against CHIKV and DENV were determined in the sera patients using capture ELISA kits EIA-3471 and EIA-3470 (DRG Diagnostics, Springfield, NJ, USA) and EI-293a-9601-G and EI 2525-9601 M (EUROIMMUN, Lübeck, Germany), respectively.

### 2.6. Induction of Anti-DENV2 and Anti-CHIKV IgG Antibodies by Inoculation of CHIKV and DENV2 in BALB/c Mice

**Figure 1:**
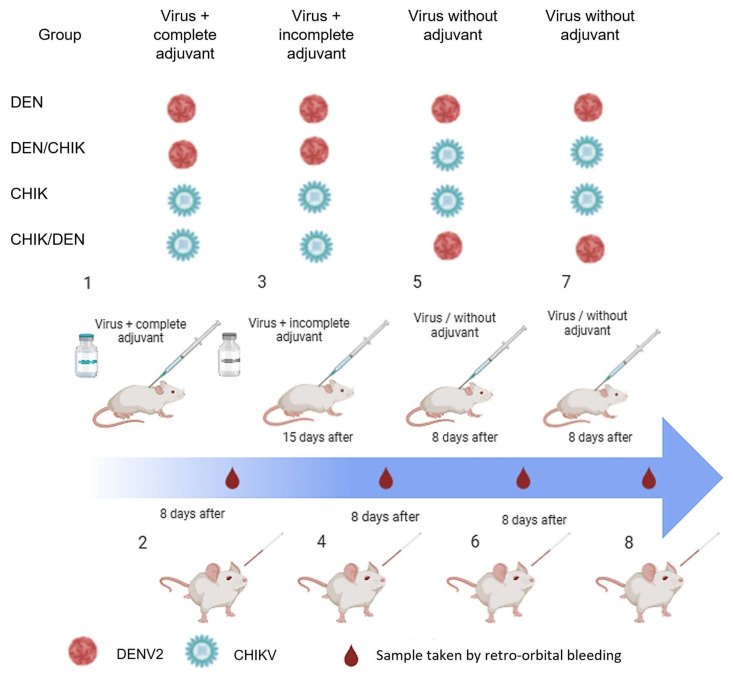
Scheme of virus inoculation in BALB/c mice. DEN: first and second inoculation with DENV2-adjuvant and two inoculations with DENV2 without adjuvant; DEN/CHIK: first and second inoculation with DENV2-adjuvant and two inoculations with CHIKV without adjuvant; CHIK: first and second inoculation with CHIKV-adjuvant and two inoculations with CHIKV without adjuvant; CHIK/DEN: first and second inoculation with CHIKV and two inoculations with DENV2 without adjuvant.

Inoculation of 6–8-week-old male BALB/c mice was performed with 30 ng of total viral protein of purified DENV or CHIKV. The mice were divided into four groups with six mice each, as follows (Figure 1):

Group DEN: The first inoculation was performed with DENV2 and a Freund’s complete adjuvant (55828, MP, Irvine, CA, USA). At 15 days, inoculation was performed again with DENV2 and Freund’s incomplete adjuvant (55829, MP). Eight days after the last inoculation, a sample was taken by retro-orbital bleeding. After eight days, the mice were inoculated again with DENV2 without an adjuvant, and after another eight days, a sample was taken in the same way. After waiting eight days again, another inoculation was performed without an adjuvant, and a sample was taken after another eight days.

Group DEN-CHIK: The first inoculation was performed with DENV2 and Freund’s complete adjuvant (55828, MP). Fifteen days later, inoculation was performed again with DENV2 and Freund’s incomplete adjuvant (55829, MP). Eight days after the last inoculation, a sample was taken by retro-orbital bleeding. After eight days, the mice were inoculated with CHIKV without an adjuvant, and after another eight days, a sample was taken in the same way. After waiting eight days again, another inoculation was performed with CHIKV without an adjuvant, and a sample was taken after another eight days.

Group CHIK: The first inoculation was performed with CHIKV and Freund’s complete adjuvant (55828, MP). Fifteen days later, inoculation was performed again with CHIKV and Freund’s incomplete adjuvant (55829, MP). Eight days after the last inoculation, the sample was collected by retro-orbital bleeding. After eight days, the mice were inoculated again with CHIKV without an adjuvant, and after another eight days, a sample was taken in the same way. After waiting for eight days again, another inoculation was performed with CHIKV without an adjuvant, and a sample was taken at eight days.

Group CHIK-DEN: The first inoculation was performed with CHIKV and Freund’s complete adjuvant (55828, MP). Fifteen days later, inoculation was performed again with CHIKV and Freund’s incomplete adjuvant (55829, MP). Eight days after the last inoculation, a sample was taken by retro-orbital bleeding. After another eight days, DENV2 was inoculated without an adjuvant, and a sample was taken in the same way eight days after the last inoculation. After waiting eight days again, another inoculation was performed with DENV2 without an adjuvant, and a sample was taken after another eight days.

Whole blood samples were centrifuged at 3500 rpm for 15 min to separate the serum. The serum samples were stored at −30 °C until use. The serum samples taken after eight days post-inoculation with the virus without adjuvant were used for the experiments.

### 2.7. Anti-DENV2 and Anti-CHIKV IgG Antibody Mouse Titration by ELISA

Ninety-six-well microplates were coated for 24 h at 4 °C with the virus (CHIKV or DENV) diluted with carbonate buffer solution (NaHCO_3_ and Na_2_CO_3_ 1 M, pH 9.6) to a final concentration of 10 µg/mL. The microwells were washed with a 0.5% Tween 20 buffer in PBS, then blocked with 5% skim milk in PBS for 1 h at 37 °C. Serum samples were diluted 1:1000 in PBS supplemented with 2% skim milk. The microwells were then washed three times. Diluted serum samples were then added to the microwells and incubated for 1 h at 37 °C. The microwells were then rewashed three times. Anti-human IgG monoclonal antibody conjugated with HRP (A0170, Sigma-Aldrich, Saint Louis, MO, USA) diluted 1:2000 in PBS–skim milk was added to the wells for 1 h at 37 °C. The microwells were then washed three times. The substrate o-phenylenediamine dihydrochloride was added, and the reaction was stopped with 2 N H_2_SO_4_ (100 µL/well); the optical density at 492 nm was measured using a microplate spectrophotometer (Thermo Fisher Scientific Multiskan MCC, Waltham, MA, USA).

### 2.8. Plaque Reduction Neutralization Test (PRNT)

A plaque reduction neutralization test (PRNT) for DENV and CHIKV was carried out using Vero E6 and Vero cells, respectively. Cells were seeded into 24-well plates at a density of 9 × 10^4^ cells/well and grown at 37 °C for 24 h.

Human serum was heat-inactivated at 56 °C for 30 min. IgG antibodies from mouse sera were purified with an NAb Protein G Spin Kit (89979, Thermo, Rockford, IL, USA). The samples were diluted with RPMI-1640 (P0870, Biowest, Nuaillé, France) and DMEM-F12 (12500062, Gibco) media at 1:5, followed by four-fold serial dilutions. DENV2 was diluted with RPMI-1640 and CHIKV viruses with DMEM-F12 media to obtain a virus infectivity concentration of 500 PFU/mL. Subsequently, 100 μL of the prepared virus was incubated with an equal volume of each diluted human serum, purified IgG mouse antibodies, or serum-free RPMI-1640 and DMEM-F12 (non-neutralization control) in duplicate at 37 °C for 1 h. The DENV2 and CHIKV suspensions and human serum mixtures were inoculated onto confluent Vero E6 and Vero cells, respectively, and incubated for 1 h with agitation every 15 min. Cells inoculated with DENV2 were gently overlaid with an overlay solution containing RPMI-1640, 2% FBS (16000044-PRO, Gibco), 100 units/mL of penicillin/streptomycin (L0022, Biowest), and 1% methylcellulose ( M0512, Sigma). Cells inoculated with CHIKV were gently overlaid with an overlay solution containing DMEM-F12, 2% FBS, 100 units/mL of penicillin/streptomycin, and 2% carboxymethylcellulose (9004-32-4, Sigma). Cells were maintained at 37 °C with 5% CO_2_. On the fifth day of incubation, DENV2-infected cells were fixed and immunostained with the anti-Flavivirus E Protein monoclonal antibody (D1-4G2-4-15, MA5-47848, Thermo Fisher). On the second day of incubation, CHIKV-infected cells were fixed and stained with crystal violet. The plaque number was counted, and PRNT_50_ was defined as a reciprocal of the highest dilution of tested serum, resulting in a 50% reduction in viral infectivity compared to the non-neutralization control.

### 2.9. Calculations and Statistical Analysis

The determination of positive human serums for anti-dengue and anti-Chikungunya IgM and IgG antibodies analyzed by the commercial kit was made according to the average absorbance of the cut-off control provided by the manufacturer. Samples were analyzed in duplicate. They were considered positive when the absorbance was greater than 10% of the cut-off value and negative when it was less than 10%.

Classification of primary and secondary dengue infection was done according to WHO criteria using IgM/IgG ratios. Primary dengue infection was defined for an IgM/IgG ratio greater than 1.2 (diluted serum 1:100) and secondary infection if the ratio was less than 1.2 [23].

Data analysis from the homemade ELISA for anti-dengue and anti-chikungunya IgG antibodies was performed by determining arbitrary units (AU) calculated using the following formula: AU = (OD of sample − OD of blank)/(OD of blank), where OD is the optical density, such that values greater than zero were taken as positive. Samples were analyzed in duplicate.

The calculation of PRNT_50_ was performed by estimating the plaque reduction proportion for each dilution: (Pd) = 1 − (nd/n0), where nd is the number of plaques at dilution d, and n0 is the number of plaques formed when no sera are added. Titers were estimated by generalized linear regression with log-transformed dilutions and the logit function (Pd) = log (Pd/(1 − Pd)).

GraphPad Prism version 6.0 (GraphPad Software, La Jolla, CA, USA) was used for statistical analysis. The values were expressed as the mean ± SD of each study group. Data were analyzed using the Mann–Whitney test, and *p*-values < 0.05 were considered statistically significant.

## 3. Results

### 3.1. Neutralizing Capacity of Anti-Dengue and Anti-Chikungunya IgG Antibodies from Patients of the State of Veracruz, Mexico

During the 2013 epidemiological outbreak of dengue in Veracruz, Mexico, samples were taken from patients with clinical data on febrile dengue and febrile dengue hemorrhagic fever. In the serum samples from patients with febrile dengue fever (DF), anti-DENV IgM antibodies were detected in 23.26% (30/129) and in 63.16% (36/57) of samples from patients with febrile dengue hemorrhagic fever (DHF) [24]. Additionally, anti-dengue IgG antibodies were detected in 93.8% (121/129) of sera from patients with a clinical diagnosis of DF and in 96.49% (55/57) with a diagnosis of DHF [24]. In addition, during the 2015 epidemiological outbreak of dengue and Chikungunya in Veracruz, Mexico, serum samples were collected, and 18% (30/167) were positive for anti-DENV IgM antibodies. Of these samples, 7% (11/167) were also positive for anti-CHIKV IgM, and 1% (2/167) were positive for anti-CHIKV IgG antibodies (Table 1). Additionally, 84% (141/167) were positive for anti-DENV IgG antibodies, of which 28% (46/167) were positive for anti-CHIKV IgM antibodies and 5% (9/167) for anti-CHIKV IgG antibodies (Table 1).

In the 2015 samples, we identified the viral RNA for DENV and CHIKV by RT-PCR. The RNA of DENV was detected in only one sample, which was positive for serotype 2. Anti-DENV IgG antibodies were also detected. The RNA of CHIKV was detected in 88 samples. Of these samples, 14% (12/88) were positive for anti-DENV IgM antibodies, and among these, 3% (3/88) were also positive for anti-CHIKV IgM antibodies (Table 2). In addition, 86% (76/88) were positive for anti-DENV IgG antibodies; of these, 26% (23/88) were positive for anti-CHIKV IgM antibodies, and 2% were positive for anti-CHIKV IgG antibodies (2/88) (Table 2).

**Figure 2:**
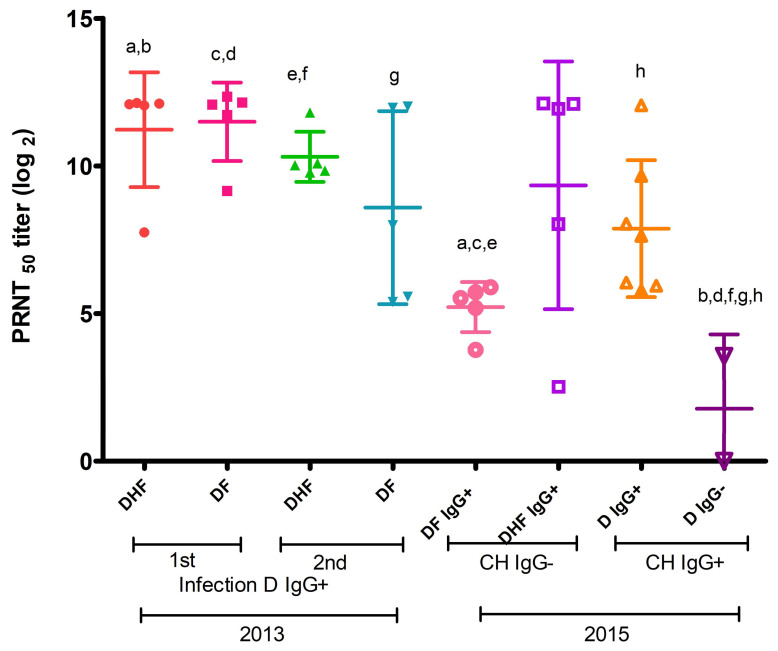
Neutralizing anti-DENV2 IgG antibodies in patient sera. PRNT_50_ titer of neutralizing IgG antibodies against DENV2 is shown. DHF: dengue hemorrhagic fever; DF: dengue febrile; D: dengue; CH: Chikungunya; 1st: primary infection; 2nd: secondary infection. One-way ANOVA, Tukey’s multiple comparisons test. Equal letters correspond to neutralization titers with statistically significant differences (statistical significance by pairs: a: *p* ˂ 0.05; b, c, e, f, g, and h: *p* ˂ 0.05; d: *p* ˂ 0.001).

The anti-DENV2 IgG neutralizing antibody titers (PRNT_50_ titers) of sera from 2013 patients (Figure 2) who presented DF and DHF in primary infection were 11.23 and 11.5 Log2, respectively. In secondary infection, the titers were 10.31 and 8.59 Log2. No statistically significant differences were found between the above groups.

We found that the primary and secondary infection anti-DENV2 IgG neutralizing antibody titers for both DF and DHF were significantly higher (Figure 2) in sera from 2013 patients than titers from 2015 anti-DENV IgG positive and anti-CHIKV IgG negative patients.

Similarly, the neutralizing activity of 2013 anti-DENV IgG antibodies from both primary and secondary infection was higher than in sera from patients with positive anti-CHIKV IgG antibodies (Figure 2).

However, the anti-DENV2 IgG neutralizing antibody titers of sera from 2015 patients positive for both anti-DENV IgG and anti-CHIKV IgG antibodies (7.88 Log2) were significantly higher than those of sera from patients positive for anti-CHIKV IgG antibodies but negative for anti-DENV IgG antibodies (1.78 Log2; *p* < 0.01) (Figure 2).

**Figure 3:**
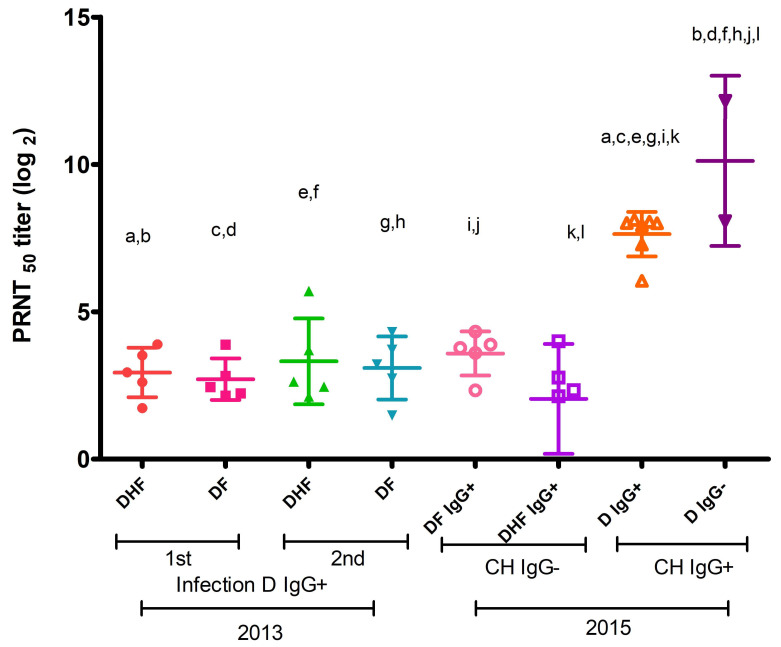
Neutralizing anti-CHIKV IgG antibodies in patient sera. PRNT_50_ titer of neutralizing antibodies against CHIKV is shown. DHF: dengue hemorrhagic fever; DF: dengue febrile; D: dengue fever; CH: Chikungunya; 1st: primary infection; 2nd: secondary infection. One-way ANOVA, Tukey’s multiple comparisons test. Equal letters correspond to neutralization titers with statistically significant differences (statistical significance by pairs, *p* ˂ 0.001).

Anti-CHIKV IgG antibody titers were detected in sera from patients with primary and secondary DF (2.712 and 3.093 Log2) and DHF (2.94 and 3.32 Log2) infection. However, no significant differences were found (Figure 3).

However, in sera from 2015 DF and DHF patients positive for anti-DENV IgG antibodies and negative for anti-CHIKV IgG antibodies, we detected neutralizing anti-CHIKV IgG antibody titers of 3.587 Log2 and 2.045 Log2, respectively. Both results suggest that anti-DENV IgG antibodies have some CHIKV-neutralizing activity.

Sera from 2015 patients (Figure 3) positive for anti-CHIKV IgG and anti-DENV IgG antibodies (7.638 Log2) and sera that were positive for anti-CHIKV IgG and negative for anti-DENV IgG antibodies (10.12 Log2) had higher titers of neutralizing anti-CHIKV IgG antibodies with respect to sera from patients positive for anti-DENV IgG antibodies from 2013 and 2015 (*p* < 0.001).

### 3.2. Induction of Anti-CHIKV and Anti-DENV2 IgG Antibodies in Sera of BALB/c Mice Inoculated with DENV2 and CHIKV

**Figure 4:**
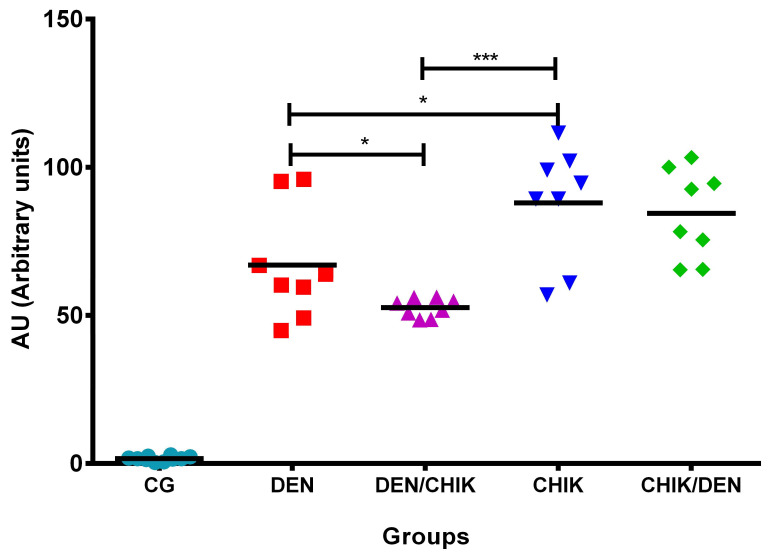
Induction of anti-CHIKV IgG antibodies in BALB/c mice. Capture ELISA assay. The CHIKV antigen was used to detect anti-CHIKV IgG antibodies. Sera dilution 1:200. CG: control group; DEN: inoculated only with DENV2; DEN/CHIK: first inoculated with DENV2 and then with CHIKV; CHIKV: inoculated with CHIKV only; CHIK/DEN: inoculated first with CHIKV and then with DENV2. Arbitrary units (AU) are reported. Mann–Whitney test, *** *p* = 0.0004, * *p* < 0.05.

The analysis of antibodies against CHIKV (Figure 4) showed that mice that were only inoculated with DENV2 presented a significant increase in anti-CHIKV IgG antibodies (66.98 ± 19.10 AU) compared with mice of the control group (1.69 ± 0.7956 AU, *p* = 0.0004) and compared to mice that were first inoculated with DENV2 and then with CHIKV (52.64 ± 3.099 AU, *p* = 0.05) (Table S1). Mice that were only inoculated with CHIKV presented a significant increase of anti-CHIKV IgG antibodies (88 ± 19.34 AU) compared to mice of the control group (1.69 ± 0.7956 AU, *p* = 0.0004), compared with mice that were only inoculated with DENV2 (66.98 ± 19.10 AU, *p* = 0.0462), and compared to mice that were first inoculated with DENV2 and then with CHIKV (52.64 ± 3.099 AU, *p* = 0.0002) (Table S1). Mice that were first inoculated with CHIKV and then with DENV2 (84.41 ± 15.15) only showed a significant increase of anti-CHIKV IgG antibodies concerning the control group (1.69 ± 0.7956 AU, *p* = 0.0004) (Table S1). These results show that IgG antibodies produced by mice that were inoculated with DENV2 react with CHIKV (cross-reactivity).

**Figure 5:**
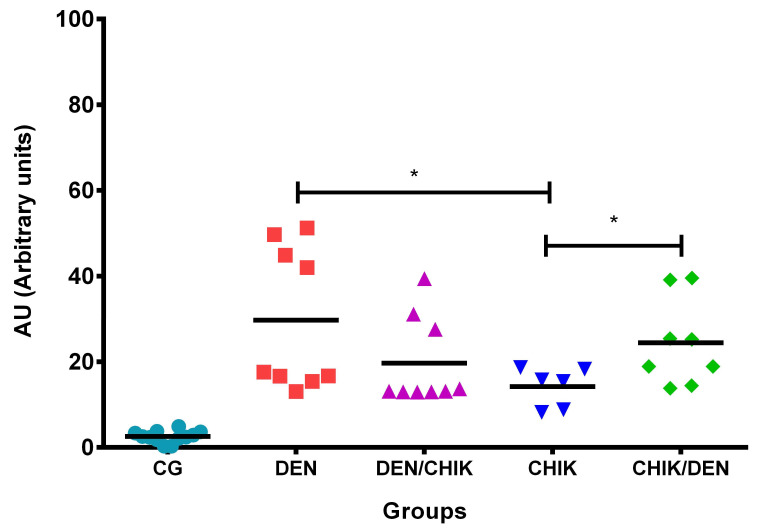
Induction of anti-DENV2 IgG antibodies in BALB/c mice. Capture ELISA assay. The DENV2 antigen was used to detect anti-DENV2 IgG antibodies. Sera dilution 1:200. CG: control group; DEN: inoculated only with DENV2; DEN/CHIK: first inoculated with DENV2 and then with CHIKV; CHIKV: inoculated with CHIKV only; CHIK/DEN: immunized first with CHIKV and then with DENV2. Arbitrary units (AU) are reported. Mann–Whitney test, * *p* < 0.05.

The analysis of antibodies against DENV2 (Figure 5) showed that mice that were only inoculated with DENV2 presented a significant increase in anti-DENV2 IgG antibodies (29.7 ± 16.63 AU) compared to the mice of the control group (2.549 ± 1.35 AU, *p* < 0.0001) and compared to mice that were only inoculated with CHIKV (14.21 ± 4.604 AU, *p* = 0.0464) (Table S2). Mice that were first inoculated with DENV2 and then with CHIKV showed a significant increase in anti-DENV2 IgG antibodies (19.67 ± 10.25 AU) compared to the mice of the control group (2.549 ± 1.35 AU, *p* < 0.0001) (Table S2); similarly mice that were only inoculated with CHIKV had a significant increase in anti-DENV2 IgG antibodies (14.21 ± 4.604 AU) compared to the mice of the control group (2.549 ± 1.35 AU, *p* = 0.0001). Interestingly, the mice that were first inoculated with CHIKV and then with DENV2 had a significant increase of anti-DENV2 IgG antibodies (24.43 ± 10.14 AU) compared to the mice of the control group (2.549 ± 1.35 AU, *p* < 0.0001) and compared with mice that were only inoculated with CHIKV (14.21 ± 4.604 AU, *p* = 0.0415) (Table S2). These results showed that IgG antibodies produced by mice that were inoculated with CHIKV reacted with DENV2, and this cross-reaction was higher when the mice were first inoculated with CHIKV and then with DENV2.

### 3.3. Determination of the Anti-CHIKV and Anti-DENV2 IgG Antibody Titer in Sera of BALB/c Mice Inoculated with DENV2 and CHIKV

**Figure 6:**
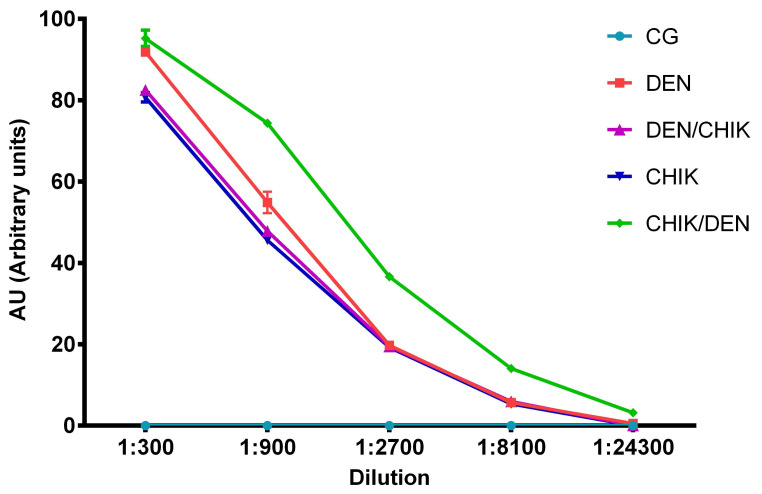
Titration of anti-CHIKV IgG antibodies obtained from BALB/c mice. Capture ELISA assay. CHIKV antigen was used to detect anti-CHIKV IgG antibodies. CG: control group; DEN: inoculated only with DENV2; DEN/CHIK: first inoculated with DENV2 and then with CHIKV; CHIKV: inoculated with CHIKV only; CHIK/DEN: inoculated first with CHIKV and then with DENV2. Titers are shown in arbitrary units (AU). Two-way ANOVA; values with *p* < 0.05 are statistically significant.

The titration of anti-CHIKV IgG antibodies (Figure 6) showed that the mice of the DEN group had higher anti-CHIKV IgG antibody titers than mice of the groups DEN/CHIK and CHIK at 1:300 and 1:900 dilutions (*p* < 0.001, Table S3). However, the titer of anti-CHIKV IgG antibodies in mice’s DEN/CHIK and CHIK groups was not statistically significantly different. The CHIK/DEN group of mice had a higher titer of anti-CHIKV IgG antibodies than the other groups of mice at all dilutions used, with different statistical significance (Table S3). These results showed that mice that were first inoculated with CHIKV and then with DENV produced a higher titer of anti-CHIKV IgG antibodies; this was even observed in the group of mice that were only inoculated with DENV, which presented a stronger IgG antibody cross-reaction with CHIKV.

**Figure 7:**
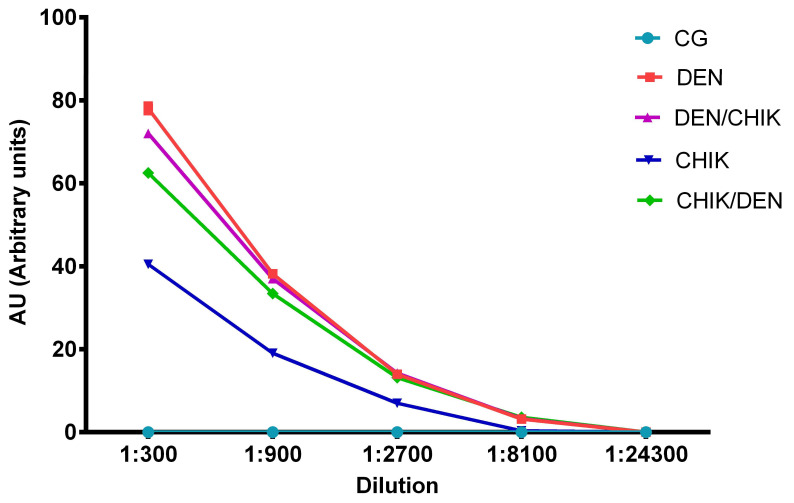
Titration of anti-DENV2 IgG antibodies from BALB/c mice. Capture ELISA assay. Here, the DENV2 antigen was used to detect anti-DENV2 IgG antibodies. CG: control group; DEN: inoculated only with DENV2; DEN/CHIK: first inoculated with DENV2 and subsequently inoculated with CHIKV; CHIK: inoculated only with CHIKV; CHIK/DEN: inoculated first with CHIKV and subsequently with DENV2. Titers are shown in arbitrary units (AU). In two-way ANOVA, values with *p* < 0.05 were statistically significant.

According to the titration of anti-DENV2 IgG antibodies (Figure 7), the DEN group of mice had higher antibody titers than the DEN/CHIK group of mice at the dilution of 1:300 (Table S4, *p* < 0.001) and more antibodies than the CHIK group of mice at dilutions of 1:300, 1:900, 1:2700 (Table S4, *p* < 0. 001), and 1:8100 (Table S4, *p* < 0.01). This result showed that the group of mice that were first inoculated with DENV2 and then with CHIKV produced IgG antibodies against DENV with almost the same titer as the group that was only inoculated with DENV2, and interestingly, the group of mice that were only inoculated with CHIKV produced anti-DENV2 IgG antibodies up to a titer of 1:2700. Interestingly, the group of mice that were first inoculated with CHIKV and then with DENV had higher anti-DENV2 IgG antibody titers than the group of mice that only were inoculated with CHIKV at the 1:300, 1:900, 1:2700, and 1:8100 dilutions (Table S4, *p* < 0.001), but the DEN group of mice produced more titers of anti-DENV2 IgG antibodies than the CHIK/DEN group of mice at 1:300 and 1:900 dilutions (Table S4, *p* < 0.001).

### 3.4. Determination of Neutralizing Capacity of IgG Antibodies in Sera of BALB/c Mice Inoculated with DENV2 and CHIKV

**Figure 8:**
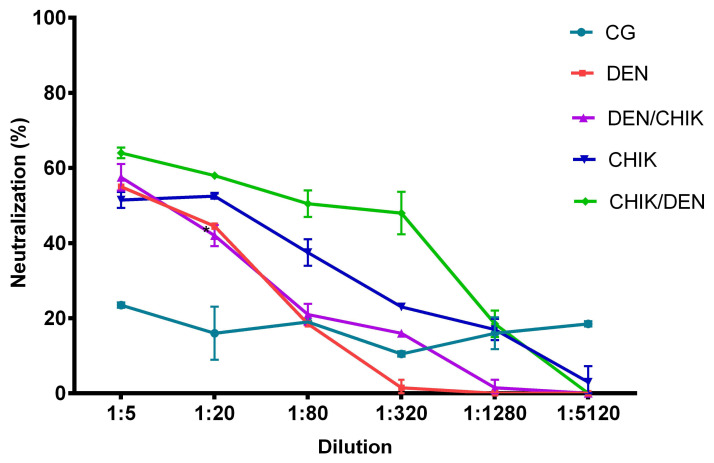
Neutralizing capacity of anti-CHIKV IgG antibodies from BALB/c mice on CHIKV-infected Vero cells. CG: control group; DEN: inoculated only with DENV2; DEN/CHIK: first inoculated with DENV2 and then with CHIKV; CHIK: inoculated only with CHIKV; CHIK/DEN: first inoculated with CHIKV and then with DENV2. The reduction percentage of PFUs is shown as 1 in 5 serial dilutions of anti-CHIKV IgG antibodies. Two-way ANOVA was applied to establish significant differences, and values of *p* < 0.05 were considered significant.

The evaluation of the neutralization capacity of anti-CHIKV IgG antibodies showed that the IgG antibodies of the mice group, which was first inoculated with DENV2 and then with CHIKV, neutralized CHIKV by 16% at a 1:320 dilution in contrast to the group of mice that were only inoculated with DENV2, which neutralized CHIKV by 1.5% (Figure 8, *p* < 0.0001).

Meanwhile, IgG antibodies of the mice that were only inoculated with CHIKV had a better CHIKV neutralization capacity compared to IgG antibodies of the mice that were only inoculated with DENV2 at the dilutions 1:20 (52.5% vs. 44.5%, *p* < 0.05), 1:80 (37.5% vs. 18.5%, *p* < 0.0001), and 1:320 (23% vs. 1.5%, *p* < 0.0001). However, it is important to highlight that IgG antibodies of the DEN group of mice had CHIKV neutralizing capacity, although it was not higher than the IgG antibodies of the CHIK group of mice, which neutralized CHIKV at up to 1:1280 (17%) and 1:5120 (3%) dilutions. Even IgG antibodies of the CHIK group of mice showed better CHIKV neutralization than IgG antibodies of the DEN/CHIK group at 1:20 (52.5% vs. 42%, *p* < 0.01), 1:80 (37.5% vs. 21%, *p* < 0.0001), and 1:1280 dilutions (17% vs. 1.5%, *p* < 0.0001) (Figure 8).

Interestingly, IgG antibodies of the mice group that was first inoculated with CHIKV and then with DENV2 had a greater CHIKV neutralization capacity than the other study groups; it neutralized CHIKV by 48% up to a dilution of 1:320 (DEN 1.5%, *p* < 0.001; DEN/CHIK 16%, *p* < 0.0001; CHIK 23%, *p* < 0.0001); we even observed better CHIKV neutralization at the dilution of 1:1280 than the IgG antibodies of the DEN/CHIK group (18.5% vs. 1.5%, *p* < 0.0001). These results showed that the cross-reactivity between both viruses generated neutralizing antibodies but that the change from CHIKV to DENV2 generated a better neutralizing anti-CHIKV IgG antibody response.

**Figure 9:**
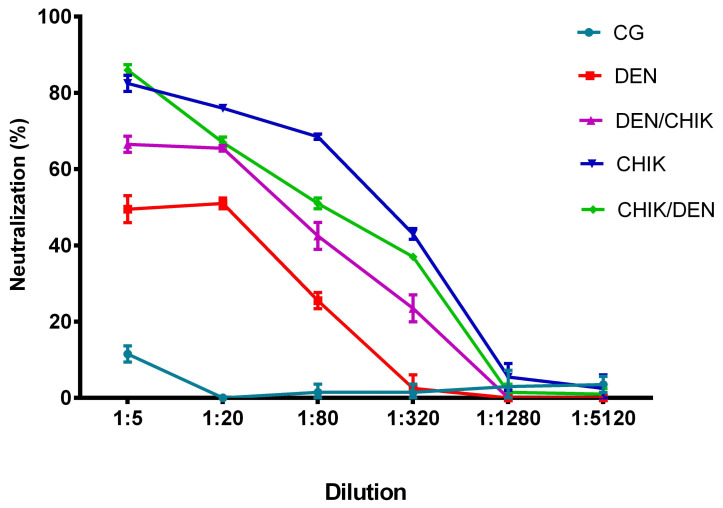
Neutralizing capacity of anti-DENV2 IgG antibodies from BALB/c mice on DENV2-infected Vero E6 cells. CG: control group; DEN: inoculated only with DENV2; DEN/CHIK: first inoculated with DENV2 and then with CHIKV; CHIK: inoculated only with CHIKV; CHIK/DEN: first inoculated with CHIKV and then with DENV2. The reduction percentage of PFUs is shown in 1:5 serial dilutions of the anti-DENV2 IgG antibodies. Two-way ANOVA was applied to establish significant differences, and values of *p* < 0.05 were considered significant.

The evaluation of anti-DENV2 IgG antibody neutralizing capacity (Figure 9) showed that IgG antibodies of the group of mice that were first inoculated with DENV2 and then with CHIKV showed a higher DENV2 neutralization than the IgG antibodies of the group of mice that were only inoculated with DENV2 up to a dilution of 1:320 (23.5% vs. 2.5%, *p* < 0.0001). Interestingly, the IgG antibodies of the group of mice that were only inoculated with CHIKV had higher DENV2 neutralizing capacity than the IgG antibodies of the group of mice that only were inoculated with DENV2 and the IgG antibodies of the DEN/CHIK group of mice up to a dilution of 1:320 (43% vs. 2.5%, *p* < 0.0001; 43% vs. 23.5%, *p* < 0.0001, respectively) and compared to IgG antibodies of the CHIK/DEN group of mice at a dilution of 1:80 (68.5% vs. 51%, respectively, *p* < 0.0001). In addition, the IgG antibodies of the group of mice that were first inoculated with CHIKV and then with DENV2 had a higher DENV2 neutralization than the IgG antibodies of the DEN and DEN/CHIK group of mice up to a dilution of 1:320 (37% vs. 2.5%, *p* < 0.0001; 37% vs. 23.5%, *p* < 0.0001). These results showed that the IgG antibodies of the mice only inoculated with CHIKV had better DENV2 neutralizing capacity, and the cross-reactivity between CHIKV and DENV2 generated better DENV2 neutralizing IgG antibodies.

## 4. Discussion

The wide distribution of the *Aedes* mosquitoes in tropical and subtropical regions has facilitated the transmission of dengue virus (DENV) and Chikungunya virus (CHIKV) in the same geographical areas [25]. Typical symptoms caused by CHIKV infection include fever, headache, myalgia, rash, and debilitating arthralgia. These symptoms are very similar to those caused by other arboviruses, especially *Flaviviruses* such as DENV, which makes diagnosis difficult [12]. According to vector competence studies, *Aedes* mosquitoes can be infected and transmit DENV and CHIKV simultaneously without seriously affecting infection, dissemination, and transmission rates in mosquitoes [26]. Thus, DENV–CHIKV infection can occur through co-infected mosquitoes or the sequential bites of mono-infected mosquitoes [15]. Cases of DENV–CHIKV co-infection have been reported worldwide, mainly during high rates of epidemic outbreaks [15,16]. In Mexico, a case of co-infection was reported during an outbreak in the Yucatan in 2014, during which it was possible to isolate DENV1 from a CHIKV-positive case [27]. CHIKV isolates in Mexico belong to the Asian lineage [28]. Co-infected patients presented more severe clinical characteristics than mono-infected cases due to the CHIKV East/Central/South African (ECSA) genotype [15].

The main neutralizing determinants are present in the E protein, specifically in domain III (ED3), in which residues incorporated in cross-reactive epitopes have been identified in different *Flaviviruses* [7,29]. ED3 peptides in different *Flaviviruses* are used for serological diagnosis and as targets for immunization because they possess epitopes with strong antigenicity that directly interact with potent neutralizing antibodies [7,30].

Antibodies against domain III are highly neutralizing only for some specific serotypes, but their neutralizing efficacy varies among the four serotypes. Antibodies directed to the fusion and bc-loop of domain II are cross-reactive but poorly neutralizing. In addition to cross-reactive and serotype-specific antibodies, quaternary structure-specific antibodies that recognize a specific structural component of the viral surface have been described [11,31].

The E2 glycoprotein of CHIKV is the main target of anti-CHIKV antibodies [31]. B-lymphocyte epitopes in patients and animal models have been identified within the E2 glycoprotein [32,33]. The amino acid sequence E2EP3 was described as one of the primary B lymphocyte epitopes among patients with Chikungunya fever and is highly conserved among most CHIKV isolates. Cross-reactivity was reported with E2PE3 and serum samples in individuals infected with arboviruses, mainly non-CHIKV *Alphaviruses, Ross River virus* (RRV), and *Barmah Forest virus* (BFV). In total, 50% of these sera neutralized CHIKV in vitro [14]. In addition, low cross-reactivity (7%) of serum samples from patients infected with *Flavivirus* and E2EP3 was observed, representing 6% of sera from patients infected with DENV, and 7% cross-reactivity between E2EP3 and sera from patients infected with non-DENV *Flavivirus*. However, these authors did not neutralize CHIKV in vitro [14].

CHIKV–DENV co-infections are clearly described [15,16]. However, it is not fully known how the host’s adaptive immune response is modified when these co-infections or primary DENV infections and subsequent CHIKV infections occur, and vice versa.

In the present study, serum samples were collected from patients with dengue and Chikungunya in Veracruz, Mexico, in 2015. Cross-reactivity was observed among these samples, with 7% found to be positive for both anti-DENV IgM and anti-CHIKV IgM antibodies and 1% positive for anti-DENV IgM and anti-CHIKV IgG antibodies. Notably, 28% of the samples were positive for anti-DENV IgG and anti-CHIKV IgM antibodies, and 5% were positive for anti-DENV IgG and anti-CHIKV IgG antibodies. However, in serum samples from dengue patients from 2013, when there were no reported cases of Chikungunya fever, we also observed cross-reactivity and neutralization with CHIKV. In serum samples from 2015 patients positive for anti-CHIKV IgG antibodies and negative for anti-DENV2 IgG antibodies, we also observed greater cross-neutralization than that observed with DENV2.

Pre-existing immunity against CHIKV could be due to asymptomatic CHIKV infections [34] or the presence of natural antibodies, which were previously proposed in mice and humans. These natural antibodies provide early protection and control the dissemination of infectious pathogens, either through direct neutralization or by activating the complement system [35,36,37]. It was proposed that these natural antibodies have neutralizing capabilities against CHIKV and control the early stages of infection in animals [38]. It remains unclear whether and to what extent natural antibodies against CHIKV exist in human populations.

The cross-reactivity and neutralization of antibodies between *Alphaviruses* were also previously described. This could be explained by the high similarity between the epitope sequences and their physicochemical properties [14]. Additionally, the possibility of undetected co-infections or asymptomatic CHIKV infections in patients infected with non-CHIKV *Alphavirus* cannot be excluded [39].

Cross-reactivity and neutralization by anti-DENV2 and anti-CHIKV antibodies in patient sera against CHIKV and DENV2 must be considered in designing diagnostic techniques and vaccines against these arboviruses in regions where they co-circulate.

In the present investigation, cross-reactivity was observed between sera from mice inoculated with DENV2 and CHIKV (DEN/CHIK group), as well as between sera of mice inoculated with CHIKV and DENV2 (CHIK/DEN group). This result could be explained by the previous inoculation with CHIKV and DENV2, respectively. However, in the DEN group inoculated only with DENV2, cross-reactivity with CHIKV was observed, and in the CHIK group inoculated only with CHIKV, cross-reactivity with DENV2 was observed. Based on the above, it can be established that the group inoculated with DENV2 (group DEN) produced anti-CHIKV antibodies. Additionally, these antibodies were increased when we inoculated initially with CHIKV and later with DENV2 (group CHIK/DEN). This result suggests that CHIKV stimulated the memory response induced by DENV2 and that DENV2 stimulated the memory response induced by CHIKV.

The mice sera in which cross-reactivity was observed also showed neutralizing activity; this suggests that anti-DENV IgG antibodies recognize and neutralize CHIKV, similarly to anti-CHIKV IgG antibodies with DENV2. When inoculated initially with CHIKV and subsequently with DENV2, the neutralizing capacity (the reduction in PFUs) for CHIKV was enhanced compared to when the mice were inoculated only with CHIKV. Anti-DENV2 IgG antibodies may recognize epitopes of CHIKV different from those recognized by anti-CHIKV IgG antibodies, which is represented as a synergistic effect.

In this study, a more significant number of anti-DENV2 IgG antibodies was found when mice were inoculated only with DENV2 compared to the group inoculated initially with DENV2 and later with CHIKV (DEN/CHIK). However, when observing the neutralization assay results, the DEN group showed a lower neutralizing capacity against DENV2 despite the greater titer of antibodies in this group. When there was a challenge with CHIKV in the DEN/CHIK group, the neutralizing capacity against DENV2 increased even though the antibody titer was lower than that in the DEN group. Furthermore, the CHIK group inoculated only with CHIKV had a better neutralizing capacity, even though the number of antibodies was lower than that in other groups. These results suggest that the antibodies generated were generally less neutralizing for DENV2 than CHIKV and that CHIKV-induced antibodies were more neutralizing for DENV2 than those induced by DENV2. Therefore, cross-reactive antibodies can neutralize both viruses to some extent. However, CHIKV antibodies induced by immune memory activation offer better neutralizing activity against DENV2 and CHIKV.

It is also essential to carry out additional studies to evaluate the cross-reactivity and neutralization capacity between antibodies and the serotypes DENV1, DENV3, and DENV4 against CHIKV and between antibodies against the different lineages of CHIKV and DENV. Indeed, differences are observable between the amino acid residues in the epitopes that serve as targets of neutralizing antibodies between DENV serotypes [7]. This phenomenon should be further investigated.

## 5. Conclusions

In serum samples from 2013 patients, we found that anti-DENV IgG antibodies cross-reacted with CHIKV and had neutralizing capacity, even though there were no reported cases of Chikungunya in Mexico. In the same way, in serum samples from patients from 2015, when there were reported cases of CHIKV in Mexico, we found that serum that were positive for anti-CHIKV IgG antibodies cross-reacted with DENV and had neutralizing capacity. In a BALB/c murine model, we found that IgG antibodies produced by mice that were inoculated with DENV2 reacted with CHIKV (cross-reactivity). Similarly, IgG antibodies produced by mice inoculated with CHIKV reacted with DENV2. This cross-reaction was more significant in the mice first inoculated with CHIKV and then with DENV2. This last group of mice produced a higher titer of anti-CHIKV IgG antibodies with neutralizing capacity; this was even observed in the group of mice that were only inoculated with DENV2, which presented a stronger IgG cross-reactive and neutralizing capacity with CHIKV. These results showed that cross-reactivity between both viruses generated neutralizing antibodies, but the exchange of CHIKV for DENV2 yielded a better neutralizing anti-CHIKV antibody response.

## Figures and Tables

**Table 1:** Serological diagnosis, *n* = 167.

Dengue, *n* (%)		Chikungunya, *n* (%)	
IgM	30 (18%)	IgM	11 (7%)
		IgG	2 (1%)
IgG	141 (84%)	IgM	46 (28%)
		IgG	9 (5%)

**Table 2:** Serological diagnosis of serum samples positive for CHIKV by RT-PCR, *n* = 88.

Dengue, *n* (%)		Chikungunya, *n* (%)	
IgM	12 (14%)	IgM	3 (3%)
		IgG	0 (0%)
IgG	76 (86%)	IgM	23 (26%)
		IgG	2(2%)

## Data Availability

The original contributions presented in the study are included in the article/Supplementary Materials, further inquiries can be directed to the corresponding author/s.

## Supplementary Materials

The following supporting information can be downloaded at https://www.mdpi.com/article/10.3390/v16071098/s1, Table S1: Induction of anti-CHIKV IgG antibodies in BALB/c mice; Table S2: Induction of anti-DENV2 IgG antibodies in BALB/c mice; Table S3. Titration of anti-CHIKV IgG antibodies obtained from BALB/c mice; Table S4. Titration of anti-DENV-2 IgG antibodies from BALB/c mice.
