# Supplementary material for: Cross-Neutralizing Anti-Chikungunya and Anti-Dengue 2 IgG Antibodies from Patients and BALB/c Mice against Dengue and Chikungunya Viruses
Source: Viruses. 2024 Jul 8;16(7):1098. doi: 10.3390/v16071098 (PMC11281444; doi:10.3390/v16071098)
Supplement: Supplementary file 1 [file viruses-16-01098-s001.zip › viruses-2914782-supplementary.pdf]

Table S1. Induction of anti-CHIKV IgG antibodies in BALB/c mice.

Groups	Mean ± standard deviation (UA)	*p*
D2 vs. CG	66.98 ± 19.10 vs. 1.69 ± 0.7956	0.0004
D2 vs. D2-CH	66.98 ± 19.10 vs. 52.64 ± 3.099	0.05
CH vs. CG	88 ± 19.34 vs. 1.69 ± 0.7956	0.0004
CH vs. D2	88 ± 19.34 vs. 66.98 ± 19.10	0.0462
CH vs. D2-CH	88 ± 19.34 vs. 52.64 ± 3.099	0.0002
CH-D2 vs. CG	84.41 ± 15.15 vs. 1.69 ± 0.7956	0.0004

Table S2. Induction of anti-DENV2 IgG antibodies in BALB/c mice.

Groups	Mean ± standard deviation (UA)	*p*
D2 vs. CG	29.7 ± 16.63 vs. 2.549 ± 1.35	< 0.0001
D2 vs. CH	29.7 ± 16.63 vs. 14.21 ± 4.604	0.0464
D2-CH vs. CG	19.67 ± 10.25 vs. 2.549 ± 1.35	< 0.0001
CH vs. CG	14.21 ± 4.604 vs. 2.549 ± 1.35	0.0001
CH-D2 vs. CG	24.43 ± 10.14 vs. 2.549 ± 1.35	< 0.0001
CH-D2 vs. CH	24.43 ± 10.14 vs. 14.21 ± 4.604	0.0415

Table S3. Titration of anti-CHIKV IgG antibodies obtained from BALB/c mice.

Groups/Dilution	Mean ± standard deviation (UA)	*p*
D2 vs. D2-CH / 1:900	54.860 ± 2.644 vs. 47.865 ± 0.374	< 0.001
D2 vs. CH / 1:900	54.860 ± 2.644 vs. 45.575 ± 0.459	< 0.001
CH-D2 vs. D2 / 1:8100	14.070 ± 0.353 vs. 5.68 ± 0.0001	< 0.001
CH-D2 vs. D2-CH / 1:24300	3.16 ± 0.155 vs. 0.085 ± 0.120	< 0.05
CH-D2 vs. CH / 1:8100	14.070 ± 0.353 vs. 5.340 ± 0.480	< 0.001

Table S4. Titration of anti-DENV-2 IgG antibodies from BALB/c mice.

Groups/Dilution	Mean ± standard deviation (UA)	*p*
D2 vs. D2-CH / 1:300	78.065 ± 1.269 vs. 72.043 ± 0.241	< 0.001
D2 vs. CH / 1:8100	3.158 ± 0.022 vs. 0.356 ± 0.044	< 0.01
D2 vs. CH-D2 / 1:900	38.189 ± 0.263 vs. 33.452 ± 0.526	< 0.001
D2-CH vs. CH / 1:8100	3.266 ± 0.175 vs. 0.356 ± 0.044	< 0.01
D2-CH vs. CH-D2 / 1:900	37.012 ± 0.131 vs. 33.452 ± 0.526	< 0.001
CH-D2 vs. CH / 1:8100	3.622 ± 0.197 vs. 0.356 ± 0.044	< 0.001
